# The function and mechanism of PSMD14 in promoting progression and resistance to anlotinib in osteosarcoma

**DOI:** 10.1186/s12935-023-03164-6

**Published:** 2023-12-05

**Authors:** Zhiyong Liu, Xin Wang, Chao Li, Ruina Zhao

**Affiliations:** Department of Orthopedics, The Affiliated Cancer Hospital of Zhengzhou University and Henan Cancer Hospital, Zhengzhou, 450008 Henan China

**Keywords:** Osteosarcoma, Resistance, PSMD14, Progression, PI3K/AKT/mTOR signaling pathway

## Abstract

**Background:**

Osteosarcoma is a rare bone malignancy that frequently affects adolescents and poses formidable obstacles in its advanced stages. Studies revealed that PSMD14 may be a viable osteosarcoma treatment target. However, PSMD14’s function and mechanism in osteosarcoma remain unknown. This study aimed to examine the function and mechanism of PSMD14 in the biological behavior of osteosarcoma and its role in anlotinib resistance.

**Methods:**

Western blotting, qRT-PCR, and immunohistochemistry (IHC) studies were used to examine PSMD14 levels. The role of PSMD14 in the malignant phenotype of osteosarcoma and its molecular pathway was explored by a series of studies, including Western blotting, cell amplification assay, transwell assay, and tumor growth. Furthermore, a series of in vitro investigations were done to determine the effect of PSMD14 on anlotinib-resistant osteosarcoma cell lines.

**Results:**

PSMD14 expression was elevated in osteosarcoma tissues compared to normal tissues. Overexpression of PSMD14 was associated with osteosarcoma patients’ pathological grade and clinical stage, and PSMD14 was an independent poor prognostic factor. PSMD14 knockdown inhibits in vitro cell proliferation, migration, invasion, and in vivo tumor growth. PSMD14 knockdown has the potential to downregulate the PI3K/Akt/mTOR pathway, which was regarded as one of the key mechanisms promoting tumor growth. PSMD14 was likewise overexpressed in anlotinib-resistant OS cell lines, and its knockdown not only reduced the proliferation, migration, and invasion of subline cells and triggered cell apoptosis. Importantly, combination therapy with anlotinib enhanced these effects.

**Conclusions:**

PSMD14 is substantially expressed in osteosarcoma and may be an independent risk factor associated with poor prognosis. It can promote tumor progression and anlotinib resistance in osteosarcoma and may promote osteosarcoma progression by modulating PI3K/AKT/mTOR signaling pathway.

## Background

Osteosarcoma is the most prevalent primary bone malignancy originating from mesenchymal tissue, with an incidence of 3–5 per million and a wide range of histological subgroups [1]. Primarily originating in the metaphysis of the long bones, it is more prevalent in children and adolescents. Despite advances in treatment, patients with advanced osteosarcoma remain dismal, with a 5-year overall survival rate of < 30% [2]. There is no consistent second-line treatment plan for advanced osteosarcoma except for normal doxorubicin and cisplatin-based chemotherapy. Increasing data demonstrated that tyrosine kinase inhibitors (TKIs), such as cabozantinib, anlotinib, and apatinib, achieved significant efficacy with minimal toxicity in advanced osteosarcoma [3–5]. However, these drugs have apparent drawbacks, such as minimal efficacy and restricted PFS. To enhance survival, additional research on osteosarcoma’s pathophysiology and drug resistance is required.

The oncogene PSMD14, also known as POH1 or Rpn11 (proteasome 26 S subunit, non-ATPase regulatory 14), has been shown to encode active deubiquitination enzymes in the ubiquitin/proteasome pathway in several cancers [6]. It also functions as a deubiquitinating enzyme, removing polyubiquitin chains from substrates and delivering them to the 20S proteasome, where substrates are successfully digested by proteolytic enzymes in the proteasome core [7]. Due to its distinctive molecular characteristics, PSMD14 can not only enhance proteolytic destruction by the proteasome. However, they also improve the stability of ubiquitination substrates, playing a significant role in several biological activities in the nucleus, including protein stability regulation, transcription, DNA repair, cell cycle, and drug resistance. Recent research has demonstrated that PSMD 14 may function as a positive or negative regulatory factor for oncogenes in human cancers [8–10]. PSMD14 expression is elevated relative to normal neighboring tissues and is related to disease progression, decreased chemosensitivity, and poor survival. Mounting evidence suggests that PSMD14 inhibitors (capzimin and thiolutin) have promising efficacy in several in vivo and in vitro tumors, including leukemia and squamous cell carcinoma [9, 11]. Recent research suggests that PSMD14 overexpression is related to a poor prognosis and may be a possible therapeutic target for osteosarcoma patients [12]. Its function and mechanism in tumorigenesis and its potential significance in drug resistance remain unknown.

In our work, we discovered that in vivo and in vitro overexpression of PSMD14 was adversely correlated with a poor prognosis in osteosarcoma; PSMD14 may induce malignant osteosarcoma characteristics by the PI3K/AKT/mTOR pathway, according to a preliminary study. Moreover, we established anlotinib-resistant osteosarcoma sublines and verified that PSMD14 was significantly expressed in the sublines and that PSMD14 knockdown could reduce the proliferation, migration, and invasion of resistant lines, as well as reverse anlotinib resistance.

## Materials and methods

### Patients and tumor specimens

Download and analyze gene expression data and relevant clinical information from the TARGET website (https://ocg.cancer.gov/programs/target) for osteosarcoma patients. Eighty-four patients were involved and split into two groups based on the PSMD14 gene expression levels: high and low. The differences in relapse-free and overall survival between the two groups were examined using the Kaplan–Meier curve. From January to August 2021, we obtained fresh tumor samples and non-neoplastic para-tumoral bone tissue samples from 13 patients with osteosarcoma hospitalized at the Department of Bone and Soft Tissue at Henan Cancer Hospital.

The bone samples were placed in liquid nitrogen immediately, and following quick freezing, they were held in a freezer at − 80 °C for RNA and protein extraction. Additionally, we gathered paraffin-embedded samples and clinicopathological characteristics (gender, age, surgical approach, and survival period) from 76 patients with complete clinical data who were pathologically diagnosed with osteosarcoma between January 2014 and January 2017. These samples were subjected to immunohistochemistry. The Henan Cancer Hospital’s institutional research ethics committee approved this study after receiving the informed permission of all participants or their duly appointed representatives.

### Immunohistochemistry (IHC) analysis

The paraffin-embedded human osteosarcoma tissue samples were sectioned at a thickness of 4 microns and, after baking at 70 ℃ for 30 min, dewaxed twice with 100% xylene each time, hydrated with gradient alcohol series, and incubated with 0.3% H_2_O_2_ in methanol to block endogenous peroxidase activity at room temperature for 15 min. Antigen recovery was done with citric acid antigen repair solution (high temperature and pressure); slices were treated with rabbit anti-PSMD14 monoclonal antibody (Abcam, 1:500, UK) at 4 °C overnight after being blocked with 10%t goat serum for 15 min. The sections were incubated with horseradish peroxide-labeled secondary antibodies (IgG-HRP, Abcam, 1:2000, UK) at room temperature for 1 h after being washed three times with PBS buffers. The samples were stained using a diaminobenzidine color development kit (Beyotime, Shanghai) following the manufacturer’s instructions, and subsequently hematoxylin was used to reverse-stain them. Last, the software Image Scope (Leica Biosystems, Germany) was used to visualize staining.

According to the percentage of stained cells (0 = negative; 1 = 6–25%; 2 = 26–50%; 3 = 51–75%; and 4 = 75–100%) and staining intensity (0 = no staining; 1 = slight staining; 2 = moderate staining; and 3 = strong staining), the specimen was graded. The score was determined using a semi-quantitative scoring method, with the low-expression group divided into products ranging from 0 to 6, and the high-expression group divided into products ranging from 7 to 12.

### Cell culture and reagents

Human OS cell lines (MG63, U2OS, 143B, and Saos-2) and normal human osteoblasts (NHOst and hFOB 1.19) were obtained from the Chinese Academy of Sciences. 143B cells were cultured in Dulbecco’s modified Eagle’s medium (DMEM, Gibco); Saos-2, U2OS, MG63, hFOB 1.19, and NHOst cells in RPMI-1640 medium (Invitrogen, USA) containing with 10% fetal bovine serum (Gibco, USA) and 100 units/mL penicillin/streptomycin (Invitrogen, USA). All cell lines were grown in a humidified incubator under humanized conditions (37 °C, 5% CO_2_). Anlotinib (AL3818, S8726) was obtained from Selleckchem, dissolved in DMSO at a stock concentration of 10 mM, and frozen at − 20 °C for future use.

### Lentiviral Infection and transfection

The negative control and lentivirus harboring PSMD14 shRNAs (sense-loop-antisense) were acquired from GeneChem (Shanghai, China). The OE-PSMD14-expressing plasmids were purchased from GeneChem (Shanghai, China). To establish a stable cell line, osteosarcoma cells were infected with lentivirus for 72 h and screened with 2 µg/mL purinomycin. The sequences for shRNAs are as followed: shPSMD14-1 (5′-CAGAAGATGTTGCTAAATT-3′), shPSMD14-2 (5′-GTACTTATGACCTCAAATA-3′), and shPSMD14-3 (5′-GTTGGATACTGTCGTATTT-3′). Transfection was carried out with Lipofectamine 3000 (Invitrogen, Carlsbad, CA, USA) per the manufacturer’s protocol.

### RNA isolation and quantitative RT-PCR (qRT-PCR)

Using qRT-PCR, the relative expression level of reference genes was measured. Briefly, total RNA was isolated from cells and tissues using TRIzol (Takara, Japan) according to the manufacturer’s recommendations. Then, a PrimeScript RT reagent Kit (Takara, Japan) was used to reverse transcribe into cDNA, and RT-PCR was performed using SYBR Premix Ex Taq (TaKaRa) on a 7500 Real-Time PCR system (Applied Biosystems, USA) according to the manufacturer’s instructions. The gene-specifific primers were as follows: PSMD14 (sense: 5′-GGAGGAGGTATGCCTGGACT-3′, antisense: 5′-GGTTTTCTCCATGCTGTTTCTT-3′), GAPDH (sense: 5′-AGGGCTGCTTTTA ACTCTG-3′, antisense: 5′-CTGGAAGATGGTGATGGG-3′). GAPDH was used as an endogenous control, and each sample’s relative gene expression was calculated according to the 2^−ΔΔCt^ method.

### Cell viability determination

Cells (1–2 × 10^4^ cells/well) were seeded into a 96-well plate in a 5% CO_2_ incubator at 37 ℃ for 24 h. Cell viability was assessed using Cell Counting Kit-8 (CCK-8) (Dojindo, Japan) after 30 min of incubation, per the manufacturer’s instructions. The absorbance values of the water-soluble tetrazolium salt were assayed using a microplate reader (Molecular Devices, USA) at 450 nm.

### Colony formation

Colony formation assays could be useful for assessing cell viability; cells at plating efficiency density (600 cells/well) were placed into a 6-well plate and cultured for 10–14 days. Following two PBS washes of each well’s cells, 100% methanol was used to fix the attached cells for 20 min. Finally, the cells were visualized by 0.5% crystal violet staining, and the colonies (> 50 cells) were manually counted with an inverted microscope (Nikon).

### TUNEL assay

For apoptosis assays, the TUNEL assay was employed to evaluate cell apoptosis ratio using a cell death kit (Beyotime, Shanghai) according to the instructions, and LSM 880 laser scanning confocal microscope (Zeiss, Germany) was used to image and analyze each slide.

### Western blot analysis

According to the manufacturer’s instructions, the total cell protein was extracted using the RIPA Lysis Buffer Kit (Biotechnology, CA). Equal amounts of protein from each sample were resolved on polyacrylamide gels with a sodium dodecyl sulfate content of 10% before being transferred to polyvinylidene fluoride membranes (Millipore, USA). The membranes were treated with certain primary antibodies overnight at 4 °C after being blocked with 5% skim milk for 1 h at 37 °C. The membranes were incubated with the proper secondary antibodies at 37 °C for 1 h the next day, following three PBS washes. The primary antibodies used were: anti-PSMD14 (1:500, Abcam), anti-GAPDH (1:10,000, Abcam), anti-PI3K (1:500, Affinity biosciences), anti-p-PI3K (1:10,000, Affinity biosciences) anti- AKT (1:10,000, Abcam), anti- p-AKT (1:10,000, Abcam), anti-mTOR (1:1000; CST), anti- p-mTOR (1:1000, Abcam), anti-Vimentin (1:2000, Abcam) and anti-Snail (1:1000, CST). An enhanced chemiluminescence kit (ECL, Germany) was used to identify protein bands, and GAPDH was used as an internal standard to gauge the protein’s intensity.

### Wound-healing assay

Single-cell suspensions were seeded into 6-well plates and then allowed to grow at 37 °C until confluence levels greater than 90% were achieved. A line was scratched with a sterile 10-µL pipette tip across the center of the cell monolayer. Then, the cells were washed and cultivated for an additional 36 h, and plate images of the gaps were obtained under a microscope at 0 and 36 h of the experiment. The cell migration rate of each sample was analyzed using Image J software.

### Transwell assay

Cells were plated in a Transwell chamber (Corning, Inc., USA) with 8 μm wells and a gel matrix (BD Biosciences, USA) for the invasion and migration assay. The technique was streamlined: plasmid-free lentivirus-infected cells were employed as negative controls, and 40 µL of diluted VitoGel 3D-hydrogel (PeproTech, USA) was added to the upper chamber in the invasion assay. Briefly, 200 µL osteosarcoma cells were added to the chamber at a concentration of 3 × 10^4^. About 600 µL of a medium containing 20% FBS was introduced into the chamber. After 36 h of incubation at 37 °C, migratory or invasive cells were fixed with absolute methanol for 5 min, washed twice with water, and stained with 0.1% crystal violet. After closure, cells were imaged and counted using an inverted microscope in three randomly selected statistical fields.

### Establishing of anlotinib-resistant osteosarcoma cell lines

To verify the effect of PSMD14 in reversing anlotinib resistance in vivo, we first established anlotinib-resistant cells (Saos-2-R and U2OS-R) with increasing concentrations of anlotinib from 0.5 to 7 µM over 6 months. The concentration of anlotinib in the medium increased gradually at a rate of 0.5 µM until the resistant sublines stabilized. During that time, the medium changed every day. Cellular sensitivity to anlotinib among resistant sublines (Saos-2-R and U2OS-R) was determined by cell viability assay. Finally, the sublines were obtained and cultured in the medium with 10 mmol/L of anlotinib.

### Xenograft nude mouse model

To investigate the effect of PSMD14 on tumor growth in vivo in nude mice, two osteosarcoma cells were stably transfected with PSMD14 knocked down and then cultured and amplified for further study. Six weeks old BALB/c nude mice were implanted subcutaneously with two exponentially growing cells (100 µL 3 × 10^7^ cells) into the flanks of their abdomen. Caliper measurements assessed Tumor sizes every other day, and tumor volume was determined according to the formula: V = [length/2] × [width^2^]. Mice were euthanized 5 weeks after planting of tumor cells. Then the transplanted tumor was dissected, weighed, and photographed. All animal experiments were conducted under a protocol approved by the Institutional Animal Care and Use Committee of Henan cancer hospital.

### Statistical analysis

The normal distribution data were expressed in means ± standard deviation (SD). Statistical differences were analyzed between the two groups using the Student’s t-test, while one-way ANOVA was applied among multiple groups. The non-normal distribution data were represented by median and interquartile, and statistical differences were determined using non-parametric rank sum tests (Mann–Whitney U and Kruskal–Wallis H tests). Kaplan–Meier method and the log-rank test was used to assess survival analysis. The relationships between PSMD14 expression and clinicopathological parameters were evaluated by the χ^2^ test or Fisher’s exact test. GraphPad Prism 9.0 (GraphPad Software, CA, USA) was used for all statistical analyses. *P* < 0.05 was considered to be of statistical significance. *P < 0.05, **P < 0.01, ***P < 0.001, ****P < 0.0001.

## Results

### PSMD14 may be related to osteosarcoma tumorigenesis, and its overexpression predicts poor survival

**Fig. 1:**
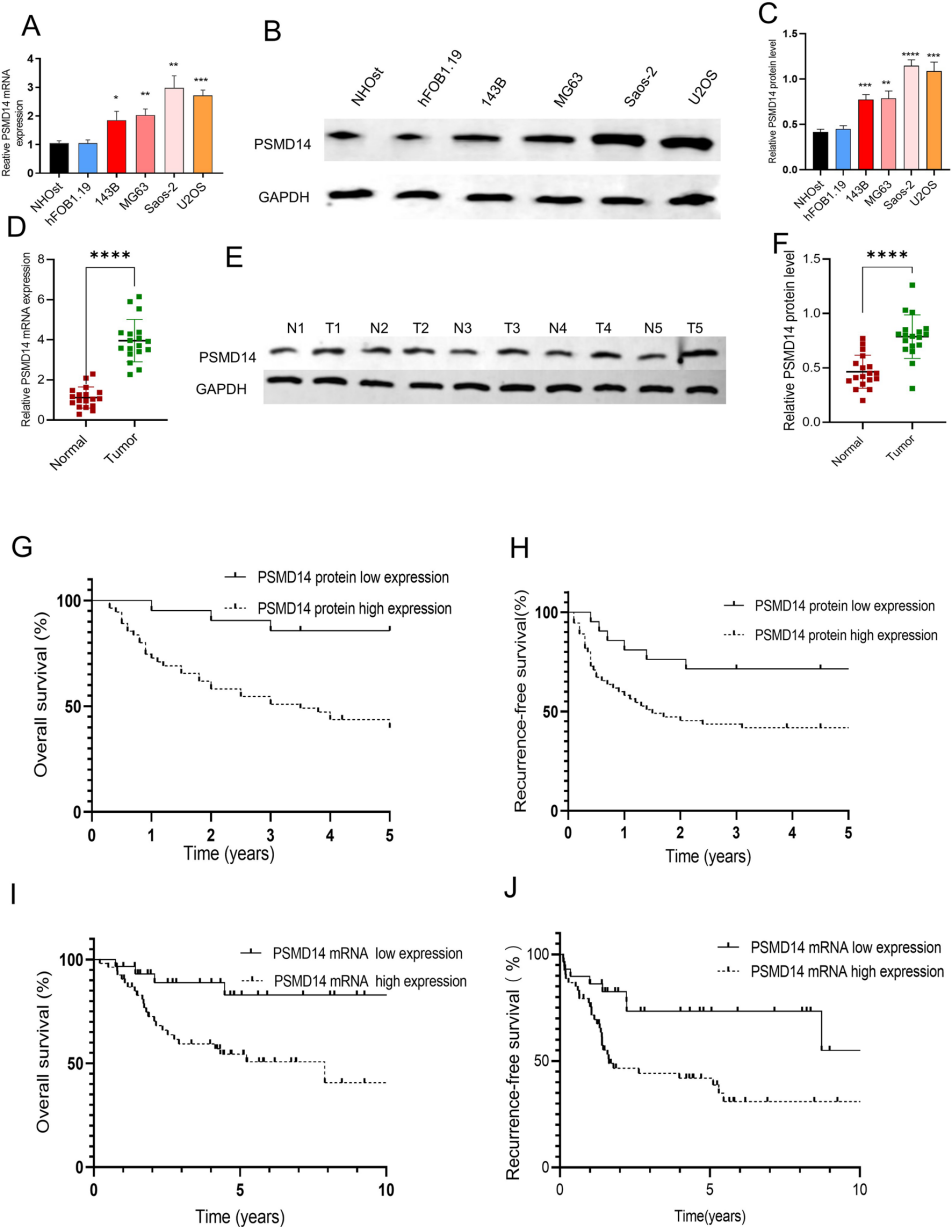
Expression of PSMD14 in patients with osteosarcoma. **A**–**C** The mRNA and protein expression of PSMD14 in normal osteoblasts and osteosarcoma cells. **D**–**F** The mRNA and protein expression of PSMD14 in osteosarcoma and paratumoral tissues. **G**, **H** The survival of osteosarcoma with different PSMD14 protein expression at our medical center. **I**, **J** The survival in osteosarcoma patients with different PSMD14 protein expression in the TARGET database

To determine the role of PSMD14 in osteosarcoma, we examined the expression of PSMD14 mRNA and protein in four human osteosarcoma cell lines (MG63, Saos-2, 143B, and U2OS) and two normal human osteoblasts (NHOst and hFOB 1.19). As shown in Fig. 1A–C, compared to normal osteoblast cells, PSMD14 was elevated at both the mRNA and protein levels in osteosarcoma cells, with Saos-2 and U2OS exhibiting the highest expression levels. Then, we investigated the mRNA and protein expression of PSMD14 in the osteosarcoma tumor tissues (“T”) and normal bone tissues (“N”) of 13 osteosarcoma patients. Consistent with the in vitro data, PSMD14 expression was higher in tumor tissues than in normal osteosarcoma tissues (Fig. 1D–F). These results revealed that PSMD14 might be crucial for osteosarcoma development, and Saos-2 and U2OS cells were chosen as osteosarcoma cell lines for more research.

Next, the association between PSMD14 expression and patient characteristics was investigated in 76 patients treated in our hospital. As indicated in Table 1, increased PSMD14 expression in tumor tissues was positively associated with the advanced clinical stage and pathological grade, and the univariate and multivariate logistic regression analysis demonstrated that PSMD14 overexpression served as an independent predictor of 5-year survival in patients with osteosarcoma (Tables 2 and 3). Further research utilizing Kaplan–Meier analysis revealed a correlation between OS patients’ high PSMD14 levels and poor overall survival and disease-free survival (Fig. 1G, H). The median overall survival in the low-expression and high-expression groups was significantly different (Fig. 1G), and the two groups also showed significantly different disease-free survival (DFS) (Fig. 1H). Osteosarcoma patients with PSMD14 overexpression had a lower disease-free survival and overall survival, as shown by the analysis of osteosarcoma data from the TARGET database using the GEPIA online software (Fig. 1I, J), which suggested that increased expression of PSMD14 predicted poor prognosis. These findings suggested that PSMD14 may play a key role in osteosarcoma progression and may serve as a new biomarker for osteosarcoma prognosis prediction.

**Table 1:** Relationship between PSMD14 expression level and clinicopathological features in 76 patients with osteosarcoma

Clinicopathological features	Number	PSMD14 expression		*P *value
		High expression (n = 51)	Low expression (n = 25)	
Gender				
Female	32	23	9	
Male	44	28	16	0.450
Age (years)				
< 18	48	30	16	0.664
≥ 18	28	21	9	
Tumor site				
Limbs	66	44	22	0.577
Others^a^	10	7	3	
Pathological subtype				
Classical	68	46	22	0.769
Others^b^	8	5	3	
Pathological fracture				
Yes	10	5	5	0.219
No	66	46	20	
Operation				
Amputation	15	8	7	0.205
Limb salvage	61	43	18	
Pathological grade				
High	65	48	17	0.004*
Moderate/low	11	3	8	
Enneking stage				
IIA/IIB	55	32	23	0.007*
III	21	19	2	

^a^Spine, pelvis, skull, scapula, etc.

^b^Periosteal osteosarcoma, paracortical osteosarcoma, telangiectatic osteosarcoma

*Fisher exact test

**Table 2:** Univariate logistic regression analysis of risk factors affecting overall survival

Clinicopathological features	Univariate logistic regression		
	HR	95% CI	*P*
Gender (male vs. female)	0.570	0.227–1.433	0.232
Age (≥ 18 vs. < 18 岁)	1.700	0.672–4.300	0.262
Site (limb vs. other^b^)	2.479	0.590–10.423	0.215
Histopathologic type (classic vs. other^a^)	3.581	0.674–19.020	0.134
Pathological fracture (yes vs. no)	1.500	0.387–5.810	0.557
Operation (amputation vs. limb salvage)	1.768	0.560–5.579	0.331
Pathological grade (high vs. moderate/low)	5.946	1.190–29.716	0.030
Enneking stage (III vs. IIA/IIB)	6.375	1.891–21.496	0.003
PSMD14 expression (high vs. low)	5.806	1.966–17.142	0.001

^a^Periosteal osteosarcoma, paracortical osteosarcoma, telangiectatic osteosarcoma

^b^Spine, pelvis, skull, scapula, etc.

**Table 3:** Multivariate logistic regression analysis of risk factors affecting overall survival

Clinicopathological features	Multivariate logistic regression		
	HR	95% CI	*P*
Pathological grade (G3 vs. G2/G1)	2.460	0.437–13.860	0.307
Enneking stage (III vs. IIA/IIB)	3.991	1.110–14.346	0.034
PSMD14 expression (high vs. low)	3.632	1.134–11.635	0.030

### PSMD14 affected the proliferation, invasion, and migration of osteosarcoma cells in vitro and tumor growth in vivo

**Fig. 2:**
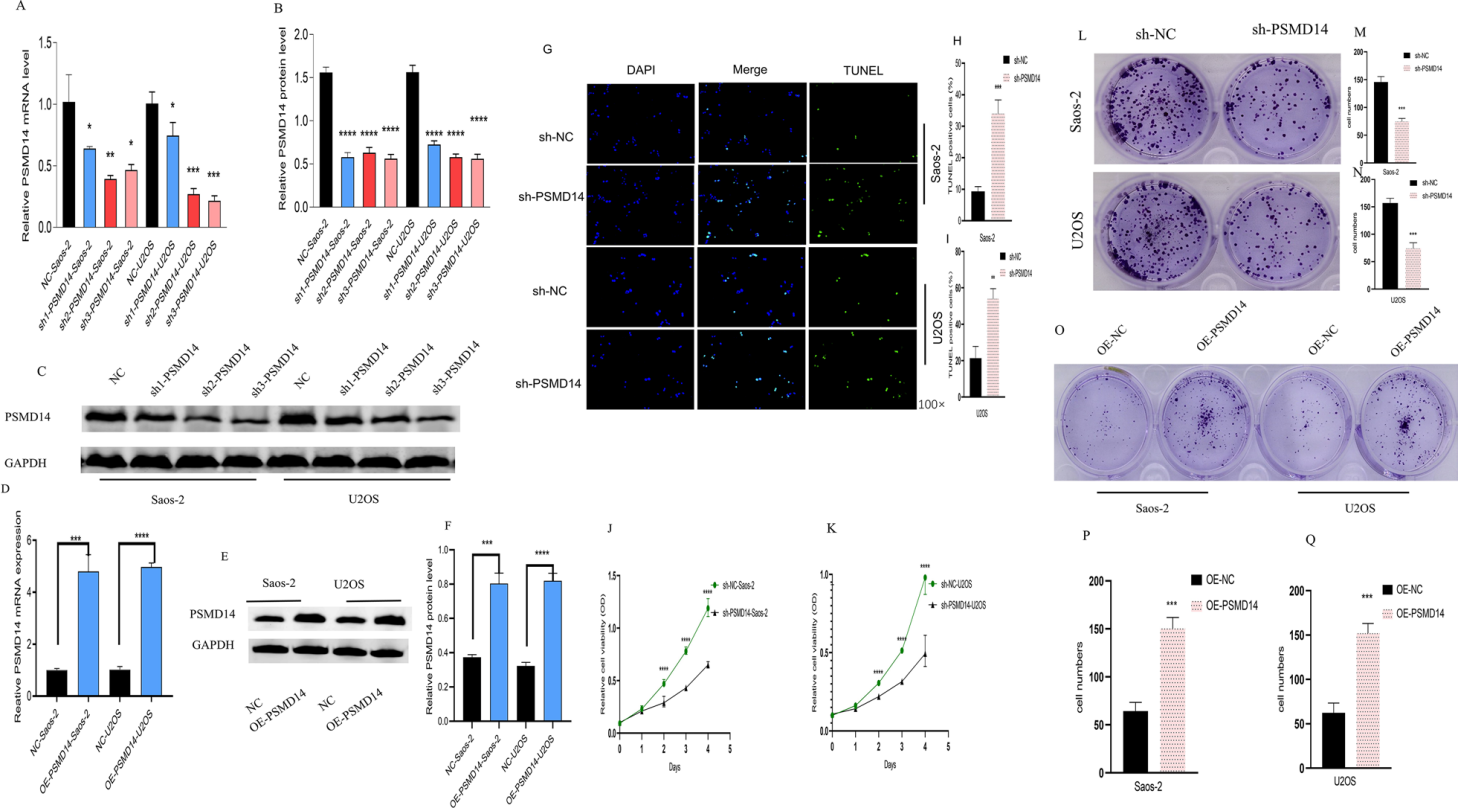
Functional effects of PSMD14 on proliferation and apoptosis. **A**–**C** The effect of PSMD14 knockdown in Saos-2 and U2OS cells. **D**–**F** The effect of overexpression of PSMD14 in Saos-2 and U2OS cells. **G**–**N** The effect of PSMD14 knockdown on apoptosis, cell viability, plate clone formation. **O**–**Q** The effect of overexpression of PSMD14 on plate clone formation

To examine the impact of PSMD14 on the malignant biological behaviors of osteosarcoma cells, we first infected osteosarcoma cells (Saos-2 and U2OS) with lentiviruses expressing shRNA to inhibit PSMD14 expression. After infection with sh2-PSMD14 and sh3-PSMD14 lentiviruses, mRNA and protein expression of PSMD14 were found to be considerably suppressed (Fig. 2A–C). To conduct further studies efficiently, sh3-PSMD14 lentivirus was chosen as the PSMD14 knockdown research object for subsequent investigations and named sh-PSMD14. Furthermore, we developed osteosarcoma cell lines (OE-U2OS and OE-Saos-2) with stable overexpression of PSMD14 (Fig. 2D–F).

To investigate the activities of PSMD14 on osteosarcoma cells, we conducted various in vitro and in vivo investigations. Firstly, we investigated the effects of PSMD14 on apoptosis and proliferation of cells. Compared to the control group, The TUNEL assay revealed that after 96 h of PSMD14 knockdown, the proportion of TUNEL positive cells increased threefold (Fig. 2G–I). CCK-8 assays demonstrated that the knockdown of PSMD14 significantly reduced osteosarcoma cell viability to around 50% of control shRNA-treated cells at 96 h (Fig. 2J, K). Colony formation assay revealed that the clones significantly decreased approximately twofold at 96 h after the knockdown of PSMD14 (Fig. 2L–N). Our results imply that PSMD14 knockdown induced osteosarcoma cell apoptosis and inhibited in vitro cell proliferation.

**Fig. 3:**
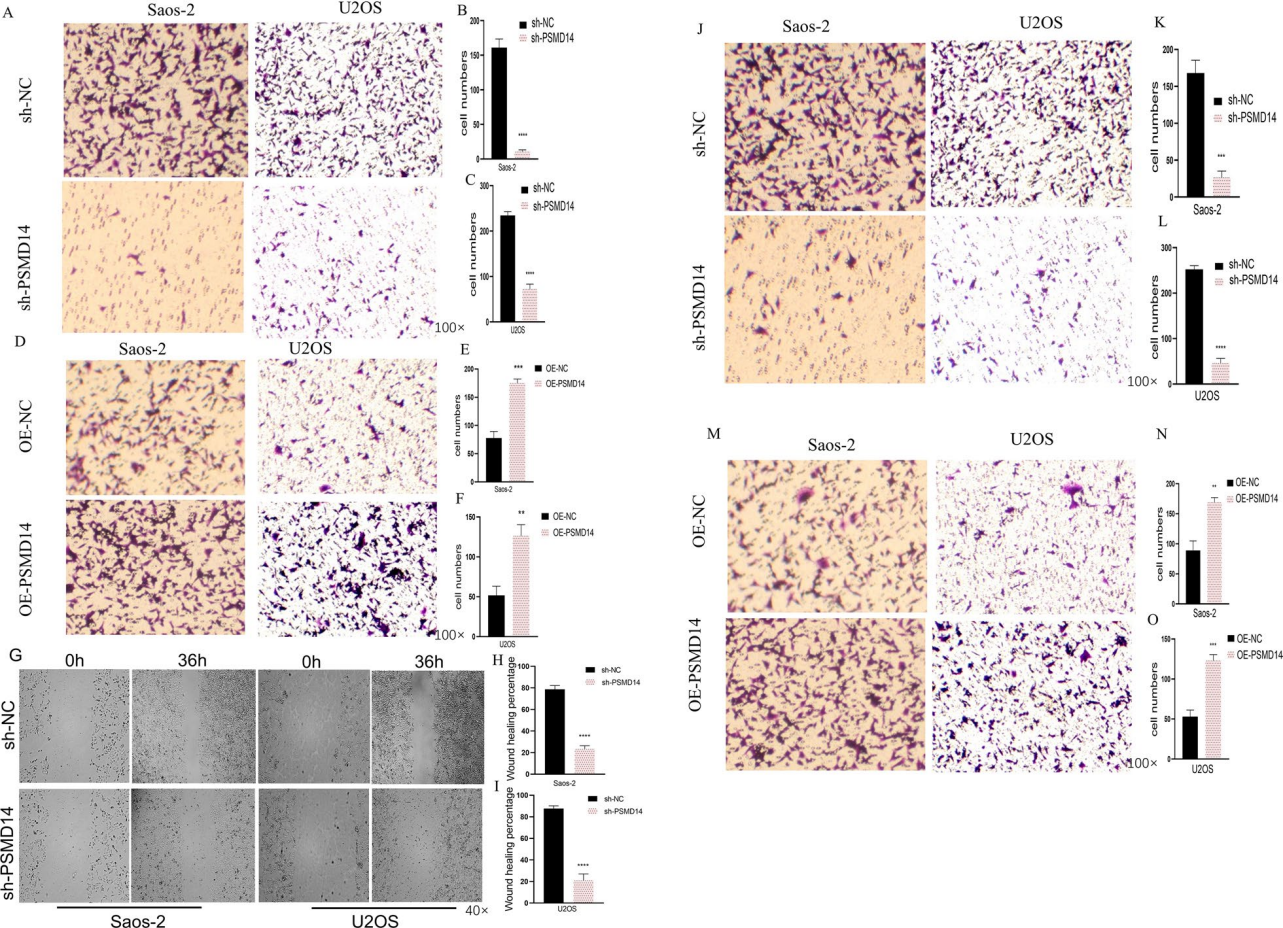
Functional effects of PSMD14 on invasion and migration. **A**–**F** The effect of PSMD14 knockdown on invasion and migration assay. **G**–**I** The effect of PSMD14 knockdown on wound-healing assay. **J**–**O** The effect of overexpression of PSMD14 on invasion and migration assay

Using transwell assays, we subsequently investigated the impact of PSMD14 knockdown on osteosarcoma cell motility. PSMD14 knockdown substantially reduced the migration (Fig. 3A–C) and invasion of U2OS and Saos-2 cells (Fig. 3D–F), and we also discovered that wound healing was impaired in both osteosarcoma cell lines (Fig. 3G–I). These results suggested that PSMD14 may considerably enhance the in vitro invasion and migratory capabilities of osteosarcoma cells.

Furthermore, we examined whether PSMD14 exerted oncogenic activities in OS cells. Based on the data of transwell assay, the migration abilities of osteosarcoma cells were significantly enhanced following PSMD14 upregulation (Fig. 3J–L), and similar results were found in invasion abilities (Fig. 3M–O). PSMD14 may play an oncogenic function in osteosarcoma cell proliferation, invasion, and migration.

**Fig. 4:**
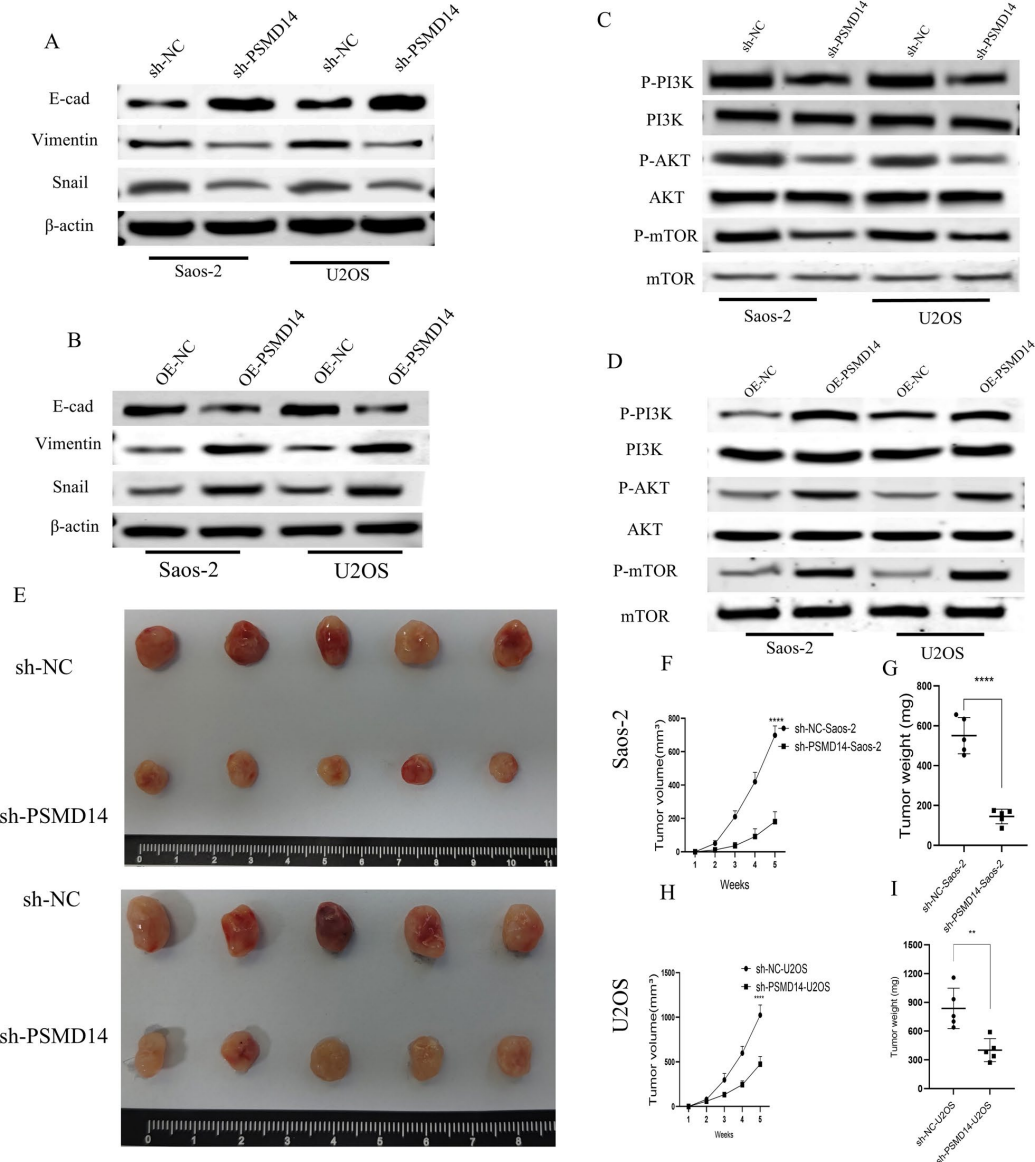
Functional effects of PSMD14 on EMT, PI3K/AKT/mTOR pathway, and tumor growth in vivo. **A**, **B** Knockdown and overexpression efficiency of PSMD14 in EMT. Knockdown and overexpression efficiency of PSMD14 in EMT. **C**, **D** The effects of knockdown and overexpression of PSMD14 on the expression of β-Actin, E-cad, Vimentin and Snail proteins. **E**–**I** The knockdown efficiency of PSMD14 in tumor growth in vivo

It has been found that aberrant activation of the epithelial–mesenchymal transition (EMT) is associated with enhanced osteosarcoma cell invasion and proliferation and a poor clinical prognosis. Using Western blot analysis, we investigated the expression of EMT-related markers in U2OS and Saos-2 cells after PSMD14 interference. PSMD14 downregulation considerably boosted E-cadherin expression while decreasing vimentin and Snail expression (Fig. 4A). PSMD14 overexpression significantly lowered the expression of E-cadherin but elevated the expression of vimentin and Snail (Fig. 4B). The data revealed that PSMD14 facilitated the EMT transition in OS cells.

To further evaluate the effect of PSMD14 on osteosarcoma cells in vivo, U2OS and Saos-2 cells from the control group and PSMD14 stable knockdown group were respectively injected subcutaneously into female BALB/c nude mice to establish a xenograft model. After 5 weeks, as depicted in Fig. 4E, the volume of the two PSMD14 knockdown groups was considerably lower than that of the control groups (Fig. 4F and H). Compared to the control groups, the weight of the two PSMD14 knockdown groups was considerably lower (Fig. 4G, I). These results indicated that stable PSMD14 knockdown also greatly suppressed osteosarcoma development.

### PI3K/Akt /mTOR acts as the downstream substrates of PSMD14, leading to tumor progression

PI3K/Akt/mTOR has been linked to carcinogenesis and progression of cancer. To determine if PSMD14 acts on PI3K/Akt/mTOR in osteosarcoma, the activation of the PI3K/Akt/mTOR pathway in upregulated and down-regulated osteosarcoma cells (U2OS and Saos-2) was evaluated. It was revealed that compared to control groups, the expression of p-mTOR, p-Akt, and p-PI3K significantly decreased in PSMD14 knockdown groups and significantly raised in PSMD14 overexpression groups. In contrast, PI3K, Akt, and TOR expression did not differ significantly between PSMD14 knockdown and PSMD14 overexpression groups (Fig. 4C, D). These findings imply that PSMD14 may increase osteosarcoma function by stimulating the PI3K/AKT/mTOR pathway.

### Establishment and characterization of anlotinib-resistant sublines

**Fig. 5:**
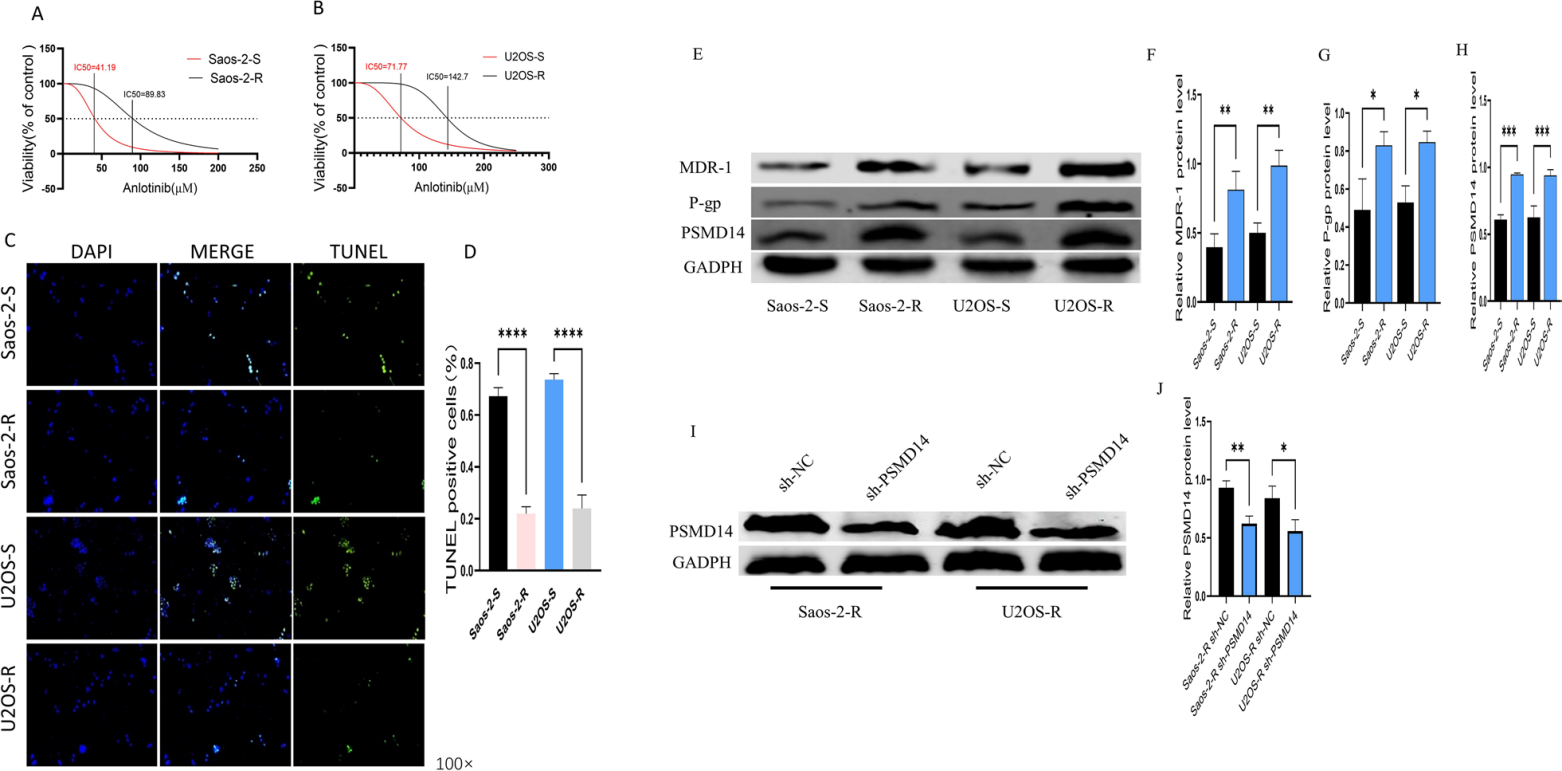
Establishment and characterization of anlotinib-resistant sublines. **A**, **B** OD values of sensitive and resistant cells with different concentrations of anlotinib for 72 h by CCK-8 method. **C**, **D** Apoptosis rates of sensitive and resistant cells. **E**–**H** The expression of MDR-1, P-gp and PSMD14 in sensitive and resistant cells. **I**, **J** The effect of PSMD14 knockdown on the expression of PSMD14 protein in resistant cells

TKI medicines for osteosarcoma were scarce in China; nevertheless, our earlier research indicated that anlotinib might be a viable target medication with a PFS of about 4 months [1]. Considering the osteosarcoma acquired and innate resistance, we evaluated the role of PSMD14 in the progression of osteosarcoma cells resistant to anlotinib. To do so, we first established anlotinib-resistant osteosarcoma cells with increasing concentrations of anlotinib, then treated sensitive cells (U2OS and Saos-2) and resistant cells (U2OS-R and Saos-2-R) for 24 h with anlotinib at varying concentrations. As demonstrated in Fig. 5A, B, the sensitive cells (U2OS and Saos-2) were more responsive to anlotinib treatment than the resistant cells when cell viability was measured using the CCK-8 assay, with a half-maximal inhibitory concentration (IC50) of 26.84 and 81.77 nmol/L for U2OS and U2OS-R, and 47.05 and 142.7 nmol/L for Saos-2 and Saos-2-R, which was similar with the findings of prior research. Using western blot analysis, we found that multi drug resistance-1 (MDR-1), P-glycoprotein (P-gp), and PSMD14 protein expression levels were considerably elevated in anlotinib-resistant cells compared to sensitive cells (Fig. 5E–H).

Next, we constructed stable resistant cells (Saos-2-R and U2OS-R) with lentiviruses expressing shRNA to inhibit PSMD14 expression, and detected significantly reduced PSMD14 expression in shRNA-treated resistant cells (Fig. 5I, J). Moreover, we treated osteosarcoma parent cells and resistant sublines with 5 µM anlotinib. TUNEL assays revealed that the rate of anlotinib-induced apoptosis was lower in resistant cells than in sensitive cells (Fig. 5C, D). These results suggested we effectively established anlotinib-resistant osteosarcoma cell lines for further study.

### Knockdown of PSMD14 resensitization of anlotinib-resistant sublines to anlotinib

**Fig. 6:**
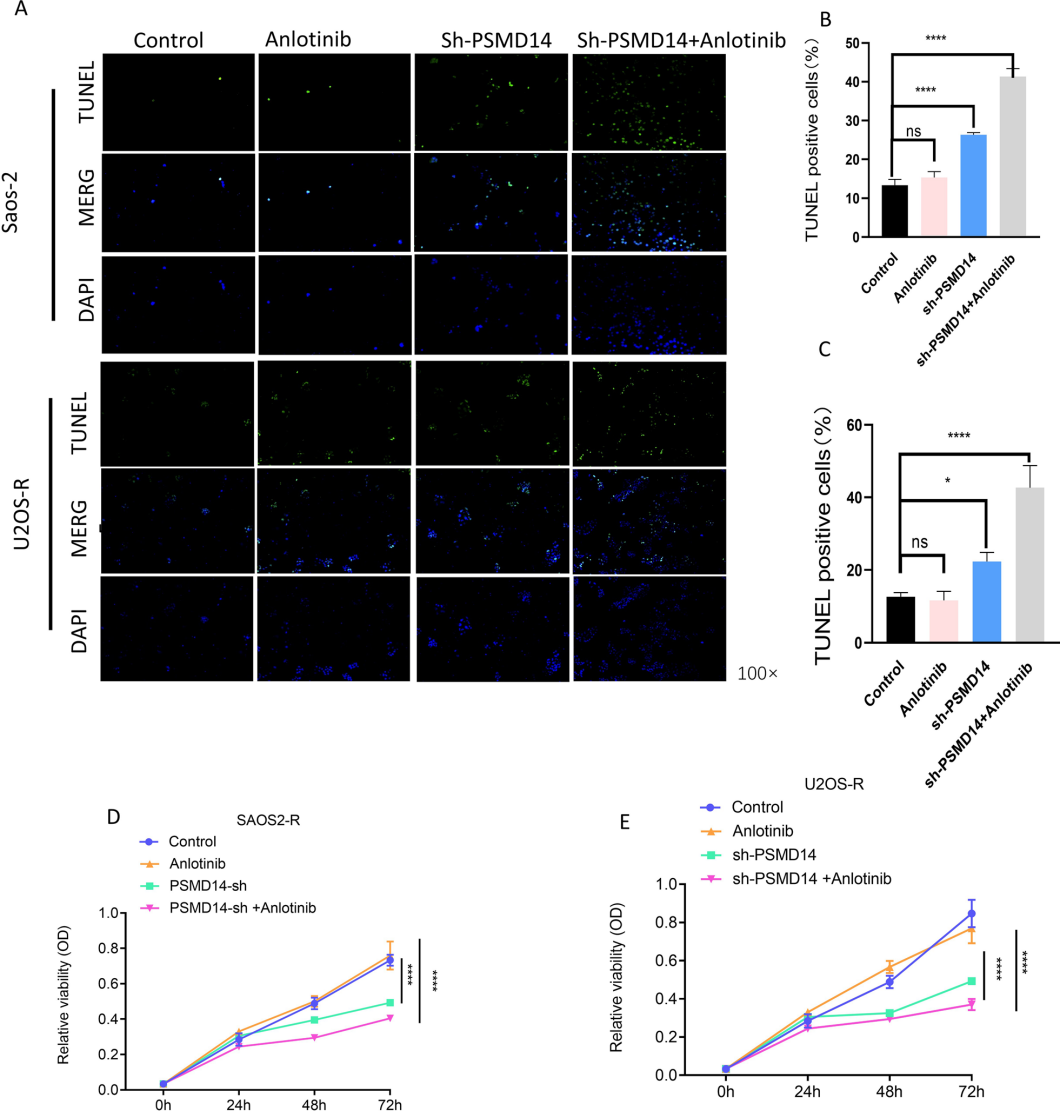
Functional effect of PSMD14 on proliferation and apoptosis anlotinib-resistant osteosarcoma sublines. **A**–**C** The apoptosis rate of resistant sublines control groups, anlotinib group, PSMD14 knockdown group and PSMD14 combined with anlotinib group. **D**, **E** The cell viability of resistant sublines control groups, anlotinib group, PSMD14 knockdown group and PSMD14 combined with anlotinib group

**Fig. 7:**
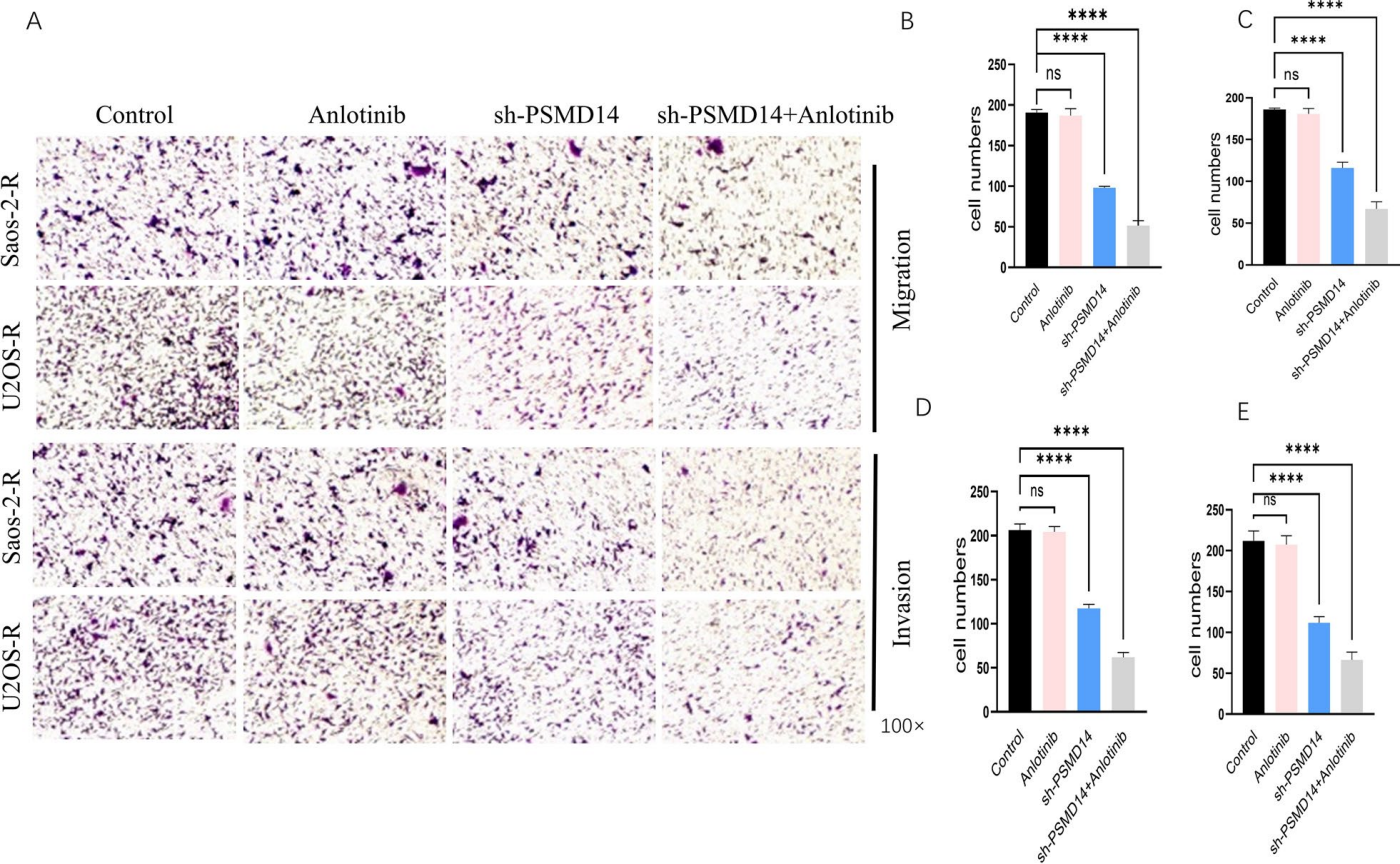
Functional effect of PSMD14 on migration and invasion in anlotinib-resistant osteosarcoma sublines. **A**–**C** The migration of resistant sublines control groups, anlotinib group, PSMD14 knockdown group and PSMD14 combined with anlotinib group. **A**, **D**, **E** The invasion of resistant sublines control groups, anlotinib group, PSMD14 knockdown group and PSMD14 combined with anlotinib group

To investigate if inhibiting PSMD14 contributes functionally to anlotinib resistance, anlotinib was used to treat OS-R (osteosarcoma resistant to anlotinib) and PSMD14-knockdown OS-R cell lines. Several functional investigations, including cell proliferation, apoptosis, invasion, and migration, were undertaken in vitro. TUNEL assays were utilized to assess cell apoptosis, and it was discovered that anlotinib did not affect the OS-R. However, PSMD14 knocking down raised apoptotic rates, and these rates were further elevated in cells treated with a combination of anlotinib and PSMD14 knocking down (Fig. 6A–C). The CCK-8 cell viability assay revealed that resistant cells treated with PSMD14 knockdown inhibited cell viability significantly compared to those treated with the control. In contrast, anlotinib had no significant effect on resistant cells. However, combining anlotinib and PSMD14 knockdown caused the most efficient cell viability inhibition (Fig. 6D, E). Similar results were observed in cell migration and invasion. The transwell experiment revealed that anltoinib had no significant influence on OS-R’s migration and invasion capabilities, whereas the combination of anlotinib and PSMD14 knocking down led to the most effective suppression (Fig. 7A–E). Thus, these results suggested that PSMD14 knockdown inhibits the malignant biological behaviors of osteosarcoma-R cell lines and reverses the inhibitory impact of anlotinib on the resistant cells’ malignant biological behaviors.

## Discussion

Osteosarcoma is the most common primary malignant bone tumor type in children and adolescents. The prognosis for patients with advanced disease is poor, with a 5-year OS of < 30%. Even though significant advances have been made in osteosarcoma bioinformatics and treatment, the persisting difficulties of tumor progression and medication resistance necessitate the development of novel techniques. Mounting evidence suggests that PSMD14 was crucial in promoting disease progression and drug resistance in several tumors, including colorectal and multiple myeloma [13, 14]. In the present investigation, we discovered that PSMD14 might be an independent biomarker of poor prognosis in individuals with osteosarcoma, and interfering with PSMD14 affects the malignant phenotype of osteosarcoma cells in vitro, including apoptosis, proliferation, invasion, and migration, as well as the growth of cell grafts in vivo. In vitro, PI3K/Akt/mTOR has been identified as the downstream substrate of PSMD14. Then, we established anlotinib-resistant sublines and identified that PSMD14 expression was elevated in resistant sublines compared to parent cells. PSMD14 knockdown plus anlotinib treatment significantly inhibited the proliferation, invasion, and migration of resistant sublines compared to anlotinib or PSMD14 knockdown treatment alone. These results suggested that PSMD14 knockdown could reverse anlotinib resistance in resistant sublines. Our findings suggest that PSMD14 may be an oncogenic gene that promotes osteosarcoma tumor growth and resistance to anlotinib by the PI3K/AKT/mTOR pathway.

Recent research demonstrates that PSMD14 can operate as oncogenes or suppressor genes to influence the formation and progression of cancers by altering the activities of critical proteins, and that PSMD14 may serve as both a prognostic indicator and a possible therapeutic target [15, 16]. PSMD14 was upregulated in tumor tissues compared to para-tumor normal bone tissues in various malignancies, including non-small cell lung cancer (NSCLC), liver cancer, and lung adenocarcinoma [17, 18]. Its overexpression is positively linked with progressive characteristics of patients, like TNM stage, increasing tumor diameter, and negatively correlated with overall survival and DFS [18]. Consistent with findings in malignant tumors, PCR and western blot analysis revealed that PSMD14 expression was elevated in tumor tissue relative to normal tissue surrounding the tumor in this investigation. Then, we calculated the scores of tumor samples using IHC and evaluated the association between the expression of PSMD14 and patient characteristics to determine if PSMD14 is a survival-predictive biomarker. The findings demonstrated that the level of PSMD14 was not only substantially related to pathological tumor grade and advanced Enneking stage. Moreover, the expression of PSMD14 was an independent risk factor for worse DFS and overall survival in patients with osteosarcoma. Therefore, the results indicated that PSMD14 overexpression may be substantially associated with osteosarcoma progression.

To further study the significance of PSMD14 in osteosarcoma, we created a stable knockdown of PSMD14 expression in human osteosarcoma cell lines Saos-2 and U2OS using lentivirus-mediated shRNA. PSMD14 knockdown suppresses in vitro and in vivo cell proliferation and colony formation. In vitro, PSMD14 overexpression stimulated osteosarcoma cell proliferation. These results were consistent with prior investigations utilizing other cancer cell lines. Lv et al. [19] discovered that PSMD14 deubiquitination could be a unique post-translational inhibitor of Grb2 in HCC cells to increase proliferation, migration, and invasion in vitro and in vivo. Zhi et al. [20] reported that PSMD14 might regulate the ubiquitination and degradation of E2F1 in glioma, consequently influencing the stability of E2F1 and subsequently regulating glioma cell proliferation and tumor growth. Zhang et al. [17] discovered that suppression of PSMD14 inhibits cell proliferation, G1 arrest, and cellular senescence while boosting cell death by upregulating p21 stability and cleaved caspase-3. Sun et al. [8] discovered that PSMD14 increases the growth of ovarian cancer by reducing pyruvate kinase activity by inhibiting the deubiquitination of the pyruvate kinase M2 isoform (PKM2). Seo et al. [21] discovered that PSMD14 could be regarded as a positive regulator that can activate the BMP6 signaling pathway and increase the stability of ALK2 via deubiquitination of the K48-linked ALK2 type I receptor, which significantly enhances tumorigenesis of colorectal cancer cells and cancer stemness/chemoresistance. Wang et al. [22] demonstrated that PSMD14 might stabilize the E2F1 protein, thereby enhancing its downstream prosurvival signals, such as the production of Survivin and FOXM1, thereby promoting tumor growth in vivo.

One of the most important signaling pathways in response to insulin signals, PI3K/Akt/mTOR, plays a crucial role in regulating malignant phenotypes such as cell proliferation, differentiation, invasion, and migration and is closely associated with the occurrence and development of malignant tumors [23, 24]. Numerous studies demonstrated that numerous receptors could activate this signaling pathway, which is involved in osteosarcoma cell proliferation, apoptosis, chemosensitivity, tumor metastasis, growth, and other malignant characteristics. Liu et al. [25] discovered that LINC00968 enhanced the proliferation, migration, and invasion of osteosarcoma cells by the PI3K/AKT/mTOR pathway. Jing et al. [10] showed that PSMD14 can improve E2F1 stability by blocking E2F1 ubiquitination in head and neck squamous cell carcinoma, hence promoting cell proliferation, stemness, and chemoresistance by the E2F1/AKT/SOX2 pathway. Wan et al. [26] demonstrated that CSN5 (a deubiquitination enzyme in the JAMM family) boosted EGFR stability by decreasing EGFR ubiquitination level, activating the EGFR/PI3K/AKT signaling pathway to promote multiple features of osteosarcoma pathogenesis. Based on the above research findings, we investigated the link between PSMD14 and the PI3K/AKT/mTOR pathway. Our western blot analysis revealed that the knockdown of PSMD14 significantly decreased the protein expression levels of p-PI3K, p-AKT, and p-mTOR but did not affect total PI3K and Akt expression levels. P-PI3 K, p-AKT, and p-mTOR levels were significantly elevated when PSMD14 was overexpressed. Therefore, we hypothesized that the PI3K/AKT/mTOR pathway might be one of the main downstream pathways that promote the development and occurrence of osteosarcoma.

Due to inherent and acquired resistance to osteosarcoma, there are limited drugs after failure to the standard treatment. Our prior research has demonstrated that anlotinib is a promising osteosarcoma-targeted drug widely utilized in China for treating advanced osteosarcoma. Recent research has studied the function and mechanism of drug resistance in osteosarcoma-targeted therapy. To further understand the drug resistance and mechanism of anlotinib, we created anlotinib-resistant osteosarcoma sublines. We found that the IC50 of the drug-resistant lines was 3 times that of the parent cells, consistent with earlier studies [27]. Then, we discovered that PSMD14 could boost the proliferation, invasion, and migration of drug-resistant strains and block the apoptosis of drug-resistant strains. Knocking down PSMD14 can restore the sensitivity of anlotinib in resistant sublines and reverse the resistance of drug-resistant strains to anlotinib. This suggests that PSMD14 promotes osteosarcoma resistance to anlotinib in resistant sublines. Previous research demonstrated that deubiquitination enzyme genes play a role in developing drug resistance in malignant tumors and that interference with PSMD14 or PSMD14 inhibitors can reverse drug resistance in malignant tumors. In esophageal carcinoma [9], PSMD14 inhibitors can diminish the association between PSMD14 and Snail, promote Snail ubiquitination and degradation, limit EMT, and inhibit ESCC cell metastasis, stemness, and chemotherapy sensitivity. In head and neck squamous cell carcinoma [28], PSMD14 can influence tumor cell proliferation, drug resistance, and cell stemness by blocking E2F1 ubiquitination and degradation, boosting Akt pathway activation, and promoting SOX2 transcription. In multiple myeloma [13], phenoxline (a PSMD14 inhibitor) can impair cellular proteasome function, activate caspase cascade and endoplasmic stress response signals to promote death in multiple myeloma cells and overcome resistance to proteosome inhibitor bortezomib, proteasome inhibitors and tyrosine kinases inhibitors produce synergistic antitumor effects. In canine osteosarcoma cells, the proteasome inhibitor bortezomib decreased cell resistance to the cytotoxic agents doxorubicin and carboplatin, and the combination of bortezomib can generate a more potent cytotoxic effect than either drug alone [29]. Therefore, PSMD14 may provide a viable method for reversing osteosarcoma’s targeted drug resistance.

First, this investigation has a few limitations due to the restricted sample size and the specimens obtained from patients having surgical resection. PSMD14 requires additional validation across many disease stages and larger populations. Second, we discovered that the PI3K/Akt/m-TOR signaling pathway plays a crucial role in PSMD14-mediated tumor cell progression in OS; however, further research is required to identify the intermediate involved. Thirdly, although we have undertaken several functional investigations on the role of PSMD14 in developing anlotinib resistance in osteosarcoma, it has to be determined if this occurs by the PI3K/Akt/m-TOR pathway.

## Conclusion

Together, this study not only shown that the carcinogenic potential of PSMD14 in osteosarcoma but also investigated the potential molecular pathways by which the pathway PSMD14 contributes to OS progression and found that PSMD14 could encourage anlotinib resistance in osteosarcoma sublines that are already resistant to the drug. Therefore, this study provides a potential strategy to treat TKIs-refractory osteosarcoma.

## Data Availability

Six cell lines (Saos-2, U2OS, MG63, 143B, NHOst and hFOB1.19) were obtained from the cell bank of Chinese Academy of Sciences in Shanghai.
